# The Circular Model in Disposal with Municipal Waste. A Case Study of Slovakia

**DOI:** 10.3390/ijerph17061839

**Published:** 2020-03-12

**Authors:** Henrieta Pavolová, Roman Lacko, Zuzana Hajduová, Zuzana Šimková, Martin Rovňák

**Affiliations:** 1 Faculty of Mining, Ecology, Process Control and Geotechnologies, Technical University of Kosice, Letná 9, 04 001 Košice, Slovakia; henrieta.pavolova@tuke.sk; 2 Department of Economy, Faculty of Business Economics, University of Economics in Bratislava, Dolnozemská cesta 1, 852 35 Bratislava, Slovakia; roman.lacko@euke.sk; 3 Department of Business Finance, Faculty of Business Management, University of Economics in Bratislava, Dolnozemská cesta 1, 852 35 Bratislava, Slovakia; 4 Faculty of Mining, Ecology, Process Control and Geotechnologies, Technical University of Kosice, Letná 9, 04 001 Košice, Slovakia; zuzana.simkova@tuke.sk; 5 Department of Environmental Management, Faculty of Management, University of Prešov in Prešov Konštantínova 16, 080 78 Prešov, Slovakia; martin.rovnak@unipo.sk

**Keywords:** environment, circular economy, ANOVA, municipal waste

## Abstract

Mineral resources are life and driving force of the European Union. It is gaining awareness not only in the EU dependent on imports, but also in the world. In the context of the growing population and the growing demands of economies for natural resources, this type of material management has a significant negative impact on the environment. The main aim of the study was to justify the model of circular economy on the national level, based on the disparities between the regions of Slovak republic. To meet the objective, mostly one-factor analysis was implemented. The circular model, which is based on the partial replacement of primary raw materials by secondary ones, should, on the one hand, limit the depletion of natural resources and, on the other hand, reduce the amount of waste produced. The presented work focuses on the issue of sustainable development, which is closely related to the circular economy, and then explains the circular economy model, including the differences from the linear arrangement and possible obstacles to its implementation for the specific conditions of the Slovak republic. From the results, it is clear that the proposed circular model would be helpful to improve the inefficiencies and disparities on the regional and national level.

## 1. Introduction

Public health is a basic assumption for increasing the prosperity of the economic system. Disturbance of ecosystem balance, permanent pollution of the environment, and its impact on human development are nowadays much discussed topics in various forums.

Given that unlimited growth on a limited planet with limited resources is clearly impossible [1,2], new production-consumption patterns, that are restorative from both intention and design points of view, become increasingly important if environmental sustainability and human wellbeing are the goals [3,4]. It stresses on closing the loop in the attempts to achieve a no ‘‘leakage” system. The model has been proved to be useful in the field of Waste-to-Energy [5,6] as a means extract value of waste, minimizing the consumption of fossil fuels. With the growing concern on sustainability, environmental impacts have also started to receive considerable attention. Other authors [7] state that the inclusion of environmental impacts can alter waste allocation patterns. Life cycle assessment is commonly applied to identify waste management and treatment systems with minimum environmental impacts [4,8,9,10]. In [11], impacts of different waste separation and treatment approaches were assessed as an extension of previous techno-economic analysis [12,13,14].

The concept of the Ordinary Economy, introduced by David Pearce in 1990, deals with the interconnections of economic environmental functions. The environment not only provides values as the basis of resources and a fall in economic activity, but it is also a vital life support system. Abreu and Ceglia identified forces that lead to a shift from the current and traditional linear flow of material and energy to the circular economy. Their study suggests that the government plays an important role in building and maintaining the cyclical flow of materials and energy [15].

The term “sustainable development” first appeared in an official document signed by thirty-three African countries in 1969 under the auspices of the International Union for Nature Conservation (IUCN). In the same year, an Environmental Protection Agency was established in the United States, whose guidelines had a huge impact on the development of theories and practices in global environmental policies. It is the documents that have started to solve the problems of our planet comprehensively, globally, and there is an initial desire for efficient resource management [16]. In the course of history, individual approaches have changed, many authors have argued that there is no sustainable development, that it is only abstraction, others have brought solutions. Whether or not there exists, future generations will be able to evaluate it or not. However, it clearly needs innovation for its operation. Innovation of thinking, in approaches and in action. Circular economy is just such an innovation [7,17].

If we talk about municipal waste in relation to circular economy (CE), every European citizen produces about 500kg of waste per year, of which less than half are recycled, at 46%, 27% are incinerated, and 24% are landfilled [18]. In the EU waste management hierarchy, waste management is ranked as follows: waste prevention, preparation for re-use, recycling, and recovery, with landfilling being the last resort. The need to efficiently treat the waste is in some countries, including Slovakia, enormous [19]. However, the CE concept is also adopted by developing countries [5,20,21].

In Slovakia, millions of tons of waste are deposited every year, which unfortunately remains for future generations, not to mention the many incidents of landfill seepage into groundwaters or rivers and landfill spills [22].

CCE will represent the latest trends in waste management in the EU and fully respect the principles of the circular economy [23,24]. The circular principle “buying—reuse—reuse once more” will become a reality in the Center of the circular economy. At the same time, the CCE concept fully corresponds to the EU vision for Slovakia by 2035. The CCE takes into account the latest trends in waste management in the European Union and translates the principles of the circular economy into practice. The result is a subject without landfill waste, so-called ZERO LANDFILL model. Waste at the Center of Circular Economy is processed through sorting, mechanical treatment, pressing, packaging, and goes back to processing [25]. The non-recoverable fractions go to the Waste Recovery Facility (WRF), where it generates heat and electricity [26].

**Figure 1:**
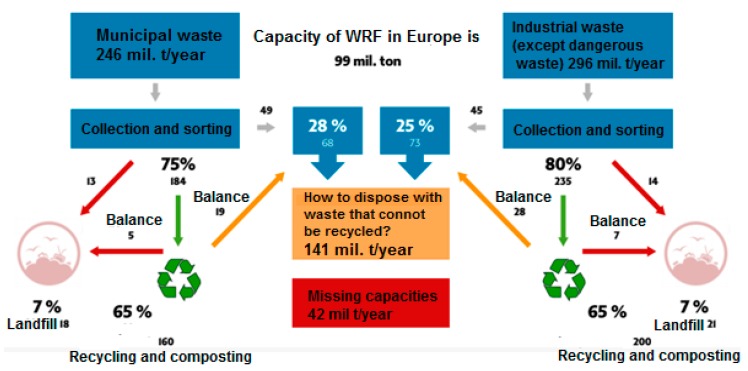
The usability of Waste Recovery Facility (WRF) capacities in the EU as well as missing capacities. Source: Based on EWIA (name of the company) model [26].

As can be seen from the picture (see Figure 1), waste recovery facilities capacities are significant within Europe, at 99 million tons. The main input to WRF is both municipal waste and industrial waste in given specific quantities (municipal waste—246 million tons per year and industrial waste—294 million tons per year). If we consider that the greater part, the greater percentage of this amount of waste will be recycled, we will always have a percentage that can be recycled no more—up to 141 million tons per year [26]. With the existing capacity of WRF, there is the problem of how to deal with 42 million tons of waste per year that cannot be recycled. Therefore, there is area for the creation of new facilities, which are also planned in Slovakia [26].

According to the above-mentioned facts and state of the art technology, the aim of this study is to propose and justify a model of circular economy suitable to the needs of Slovak republic and to other countries in the region of central Europe. For this purpose, we need to verify some research questions connected to waste management and policy of Slovak republic.

## 2. Materials and Methods

Data for this study were collected from the Statistical office of the Slovak Republic [27].

We have used graphical methods of statistical analysis. In its simplest form, the boxplot presents five sample statistics—the minimum, the lower quartile, the median, the upper quartile, and the maximum—in a visual display. Correlation coefficient used in correlation analysis is a statistical measure of the strength of a linear relationship between paired data [28]. The sign of the correlation coefficient determines whether the correlation is positive or negative.

In this article, we assess the interregional differences in regions of Slovak Republic. Thus, we need to use adequate statistical methods, which can reveal interesting facts. The normality is tested by Shapiro-Wilk Test. It is more appropriate for small sample sizes (less than 50) and the samples used in research to a lesser extent. The null hypothesis for this test is that the data are normally distributed. Alternative hypothesis is that data are not normally distributed. If the chosen alpha level is 0.05 and the *p*-value is less than 0.05, then the null hypothesis that the data are normally distributed is rejected. If the *p*-value is greater than 0.05, then the null hypothesis is not rejected. The decision for a suitable test (parametric, non-parametric) is determined by knowledge of normality and also by homogeneity of data. Normality of data was verified by Shapiro-Wilk test and homogeneity by Levene’s test. It tests the null hypothesis that the variances of the groups are the same. An alternative hypothesis is that the variances are not the same. *p*-Value being greater than 0.05 means that the variability in two conditions is about the same, so the variability in the two conditions is not significantly different (homogeneity) [29]. The statistical significance of differences in comparative analysis is tested by one-way analysis of variance (ANOVA) and Kruskal-Wallis test for independent variables and by T test or Wilcoxon signed-rank test for paired variables. ANOVA is used to determine whether there are any statistically significant differences between the means of three or more independent groups. It compares the means between the tested groups and determines whether any of those means are statistically significantly different from each other. When the assumptions of ANOVA are not met, the nonparametric Kruskal-Wallis test is used [30,31]. The paired t-test is used to compare two sets of scores that come from the same participants. This can occur when is investigating the changes in scores from one time point to another, or when individuals are subjected to more than one condition and reports if the mean of the differences is statistically significant. The null hypothesis assumes that the mean of two paired samples are equal (H0: μ1 = μ2). The alternative hypothesis assumes that the means of two paired samples are not equal (HA: μ1 ≠ μ2).

Several tests are performed to select the appropriate estimation method for panel data regression. We used the F-test for testing, whether there are panel effects in the model. If the null hypothesis is rejected, the fixed-effect method is better than pooled OLS method for the coefficient estimation. One-factor analysis was used in the statistical assessment of developmental characteristics of municipal waste recovery according to regions of Slovakia. This represents a simple form of analysis of variance, in which we assumed that the level of average of the basal sets generated by us to municipal waste recovery in individual regions of the SR depends only on one factor A.

In general, for a one-factor analysis, the model looks like this:

*Y_ij_* = *μ* + *τ_i_* + *ε_ij_, i,j* = 1, ..., *n* (1)

where

*μ*—overall average,
*τ_i_*—level effect i of factor A,
*ε_ij_*—additional random component (error)—is stochastically independent of partitioning N(0, σ^2^_ε_).

The variability was measured by the size of the deviations in the ANOVA models using the sums of squares by relationship

*SS_T_* = *SS_M_* + *SS_E_* (2)

where

*SS_T_*—is total sum of squares deviations,
*SS_M_*—is the sum of squares explained by the model,
*SS_E_*—is the sum of squares not explained by the model.

The sum of squares of residual deviations is

(3) $$SS_{E}={\sum_{i=1}^{a}\sum_{j=1}^{n_{i}}\left(y_{ij}-\bar{y_{i}}\right)}^{2}=\sum_{i=1}^{a}\left[\sum_{j=i}^{n_{i}}{\left(y_{ij}-\bar{y_{i}}\right)}^{2}\right]$$

(4) $${s_{i}}^{2}=\frac{\sum_{j=1}^{n_{i}}{\left(y_{ij}-\bar{y_{i}}\right)}^{2}}{n_{i}-1}\;\;\;\;i=1,2,\;\ldots ,a$$

## 3. Results

**Figure 2:**
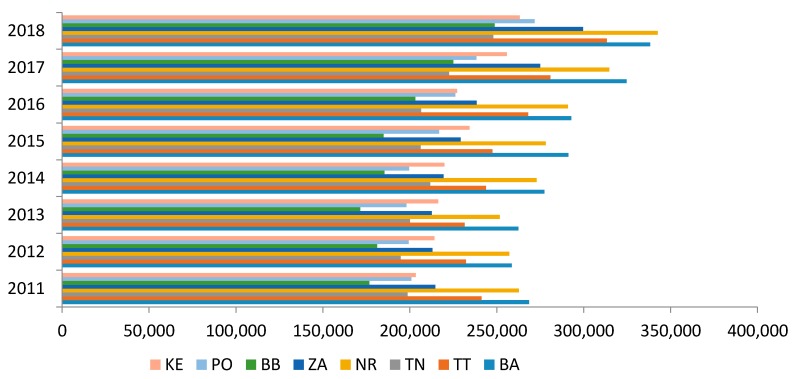
Development of municipal waste production in the regions of Slovakia. Source: Based on of data collected by Statistical Office of Slovak Republic [29]. Note: KE—Košice region, PO—Prešov region, BB—Banská Bystrica region, ZA—Žilina region, NR—Nitra region, TN—Trenčín region, TT—Trnava region, BA—Bratislava region.

The production of municipal waste in the Slovak Republic showed a fluctuating trend of development in the period under review with an average year-on-year increase of 79,741.1 t·year^−1^ and significant regional disparities. Figure 2: Here we could state that the Bratislava region had the highest production in the monitored period with an average annual production of 289,243.4 t·year^−1^ (except for 2018, when was recorded the highest production in the Nitra region) and on the contrary, the lowest production was reported in the Banská Bystrica Region with an average annual production of 229,304.1 t·year^−1^ (except 2018, when the lowest production was recorded in the Trenčín region). From a detailed analysis of year-on-year movements in municipal waste production, we found that the highest year-on-year increase in the monitored period was in the Žilina Region with a value of 12,138.1 t·year^−1^ and the lowest in the Trenčín region with an average municipal waste value of 7035.0 t·year^−1^.

**Figure 3:**
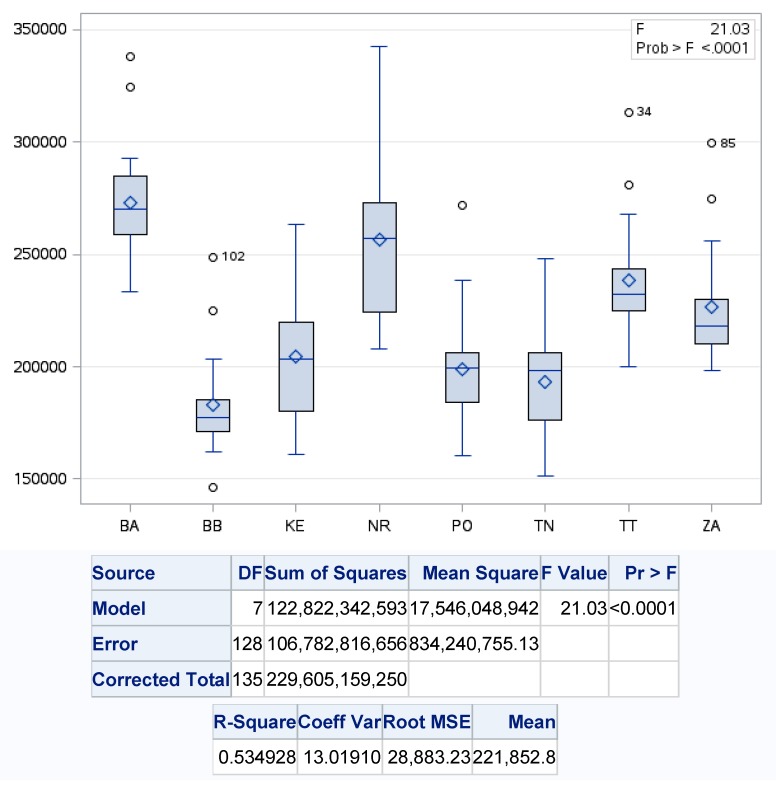
One-factor analysis of municipal waste production in the regions of Slovakia. Source: Based on of data collected by Statistical Office of Slovak Republic [29].

Based on the principles of the circular economy and accepting rather significant developmental disparities in the production of municipal waste in individual regions of Slovakia, we also examined statistically significant differences in production by individual regions of Slovakia. We can conclude from the one-factor regression analysis of the production of municipal waste in the analyzed regions of Slovakia, whose value of variability of the regression model reached the level of almost 54%, that there are significant statistical differences in the analyzed development, because of *p* value <0.001. While the biggest differences in municipal waste production are in the Nitra region (NR) and the lowest in the Banská Bystrica region (BB) (Figure 3).

**Figure 4:**
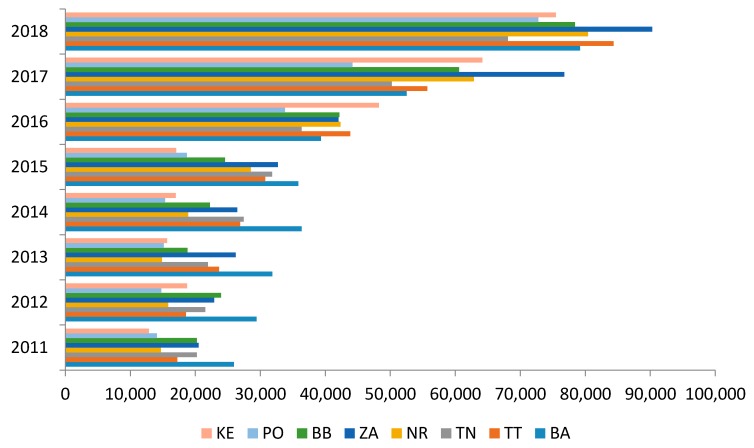
Development of the amount of separated municipal waste in the regions of Slovakia. Source: Based on data collected by the Statistical Office of Slovak Republic [29].

Given the fact that the circular model determines stable economic growth and increasing environmental quality by the efficient recovering of used materials and products, we could say that in the area of municipal waste production, the key determinant of the implementation of this model is the separation and subsequent recovery of produced municipal waste. Separation of municipal waste, in contrast to its fluctuating trend of its production, showed an increasing nature of development with an average separated quantity in Slovakia during the analyzed period of 289,253.3 t·year^−1^. It means average annual share of 14.39% per year. Based on a detailed analysis, we could state that in the years 2011–2015, the highest amount of separated municipal waste was reported by the Bratislava region (BA) with an average annual separation of 41,298.0 t·year^−1^. In 2016, the Košice region (KE) had an average annual separation of 33,636.3 t·year^−1^, and in 2017–2018 the most separated municipal waste was the Žilina Region (ZA) with the highest average separation value of 42,232.4 t·year^−1^. We have also identified outliers, which were detected by statistical software. They show that development of waste production over several years varies strongly. Differential development was recorded in the lowest values of municipal waste separation, where we found that Figure 4:

- in 2011 the inhabitants of the Košice Region separated the least,
- in 2012 and 2014 and 2016–2017, the inhabitants of Prešov region (PO) separated the least, whose average value of separation of produced municipal waste reached the level of 28,601.1 t·year^−1^, which we could also describe as the lowest average amount of separated waste the analyzed period in the regions of Slovakia,
- in 2013, the lowest value of separation showed the Nitra region (NR), whose average value of the separation of produced municipal waste reached 34,802.0 t·year^−1^,
- in 2015, the lowest value of separation showed the Košice region (KE),
- in 2018, the lowest value of separation showed the Trenčín region (TN), whose average value of separation of produced municipal waste reached 34,705.6 t·year^−1^.

The highest average share of separated municipal waste was recorded in the Banská Bystrica region (BB) at 17.51%·year^−1^ and the lowest in the Nitra Region (NR) at level 11.60%·year^−1^. The highest increase in 2018, in comparison with 2011, we found in the Košice region (KE) at level 22.36% and the lowest in Bratislava region (BA) at level 13.77%.

**Figure 5:**
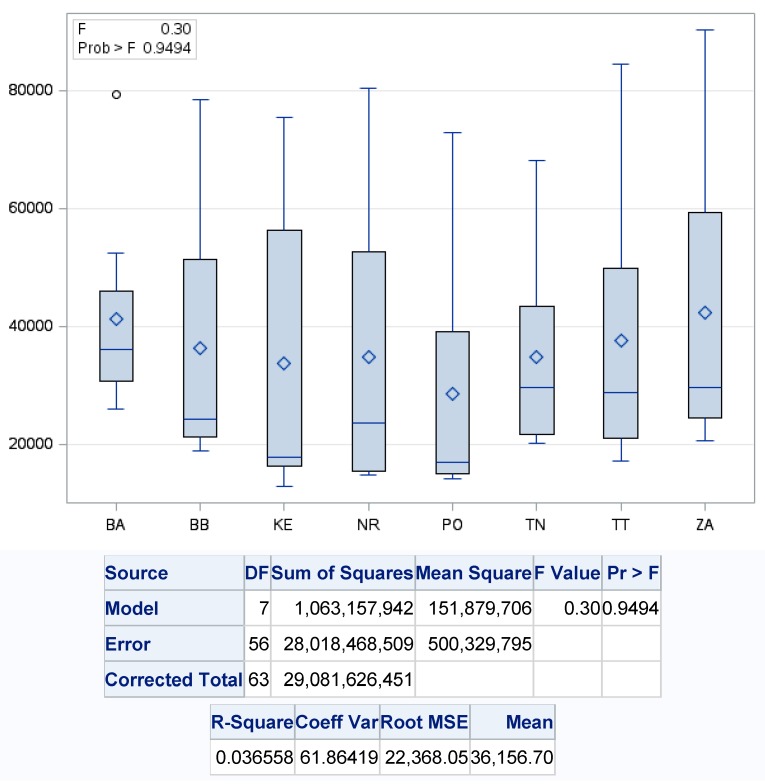
One-factor analysis of municipal waste separation in the regions of Slovakia. Source: Based on of data collected by Statistical Office of Slovak Republic [29].

We are of the opinion that when implementing the circular model to waste management, it is necessary to emphasize in particular the recovery of produced municipal waste, the primary determinant of which is the separation. This is the baseline platform for further waste recovery in accordance with the principles of a circular economy with the acceptance of green logistics, which puts the main emphasis on the return material flows in waste management, it means material recovery of waste [17]. Figure 5 shows the differences between regions in terms of sorted waste. There were no statistically significant differences in the development of separation of the municipal waste produced, since *p* value reached the level of >0.9494, with the largest variability being recorded in the Žilina region (ZA) with the maximum separation rate in 2016 at 42,036.0 t and the lowest in 2011 at the level of municipal waste separation at the level of 28,456.2 t·year^−1^. The smallest average value of the separated waste was recorded in the Bratislava region (BA) at the level of 39,757.8 t·year^−1^, in 2011 when the separated waste was 25,914.0 t and the highest in 2018, when the separated waste was 79,209.

**Figure 6:**
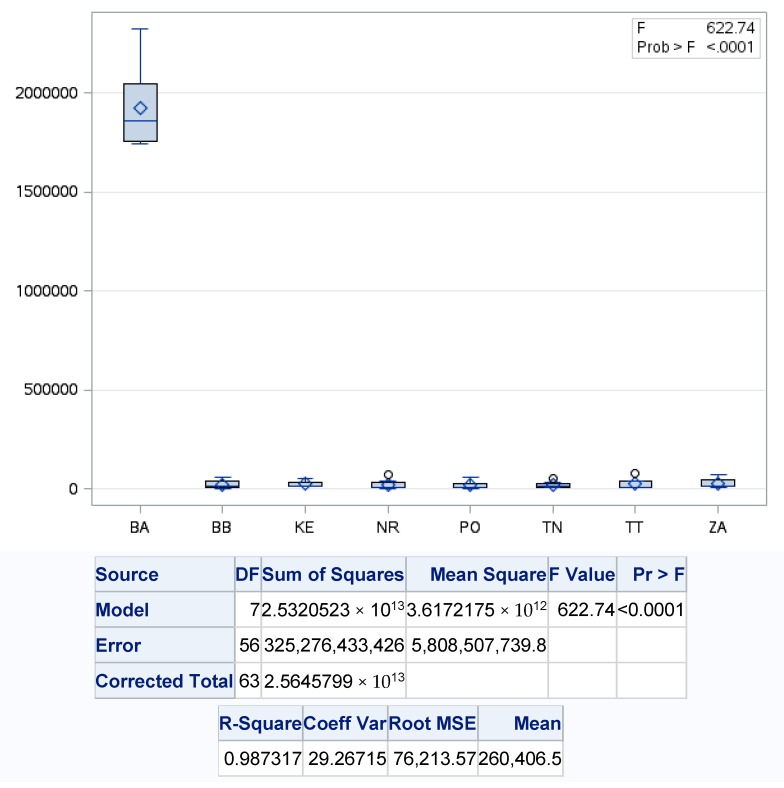
One-factor analysis of material recovery of municipal waste on the regions of Slovakia. Source: Based on of data collected by Statistical Office of Slovak Republic [29].

From the above-mentioned results of the detailed statistical analysis, in terms of the implementation of the circular model leading to the increase to the recovery of produced municipal waste, which is characterized by significant disparities in Slovakia regions, we considered that it is necessary to point out the individual forms of its recovery. One-factor regression analysis showed us significant differences in material recovery in Slovakia regions, because of *p* value <0.001, with the level of variability of regression model at almost 99%. The highest values of material recovery of municipal waste were found in the Bratislava region (BA), where the average level of recovery reached 18,728.8 t·year^−1^, with the maximum material recovery in 2018 (57,313.8 t) and the lowest in 2011 (2901.9 t) contrasting the lowest in the Trenčín region (Figure 6).

Significant disparities were also found in the case of the energy recovery of municipal waste during the analyzed period, where we could again state significant differences between the analyzed regions of Slovakia, because of *p* value showed a value of <0.001, with the level of variability of the regression model more than 54%.

**Figure 7:**
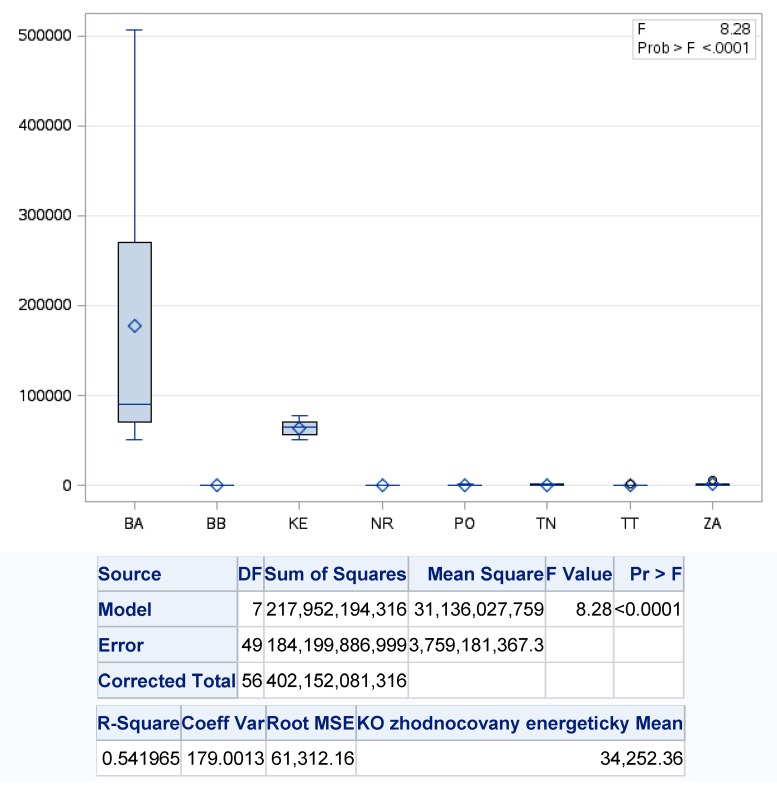
One-factor analysis of energy recovery of municipal waste on the regions of Slovakia. Source: Based on of data collected by Statistical Office of Slovak Republic [29].

The most energy recovered municipal waste was recorded in the Bratislava region (BA), where we recorded the average energy recovery at 114.27 t·year^−1^, with the lowest level of energy recovery in 2018 (83,137.8 t) and the highest in 2011. The least energy recovered municipal waste was in the Nitra region (NR), where it was only 125.6 t·year^−1^ averaged. The second region from the point of view of an efficient circular model in the case of energy recovery was the Košice Region (KE), in which approximately 76,977.1 t·year^−1^ of municipal waste was recovered on average (Figure 7).

**Figure 8:**
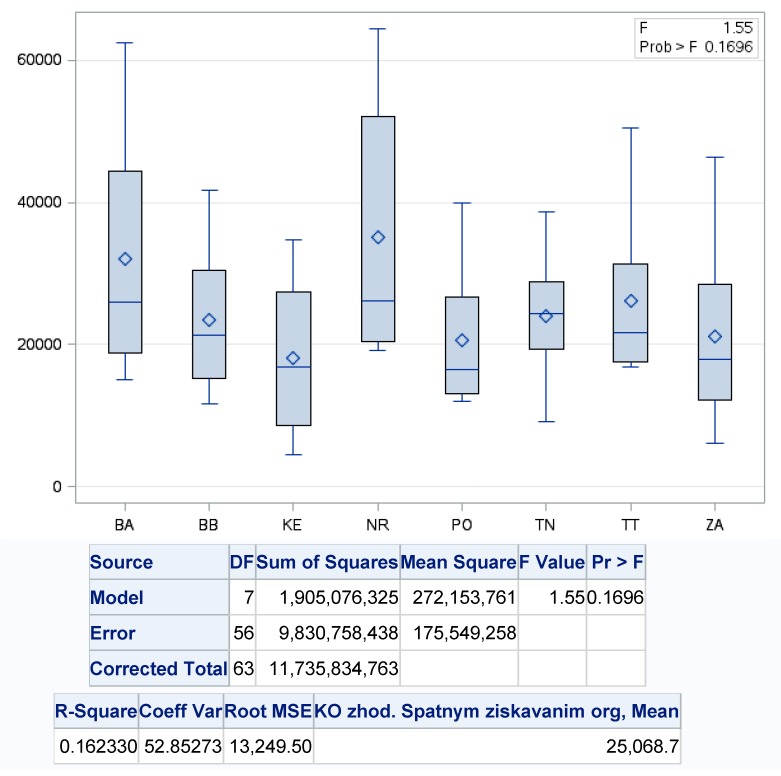
One-factor analysis of backward obtained organic materials. Source: Based on of data collected by Statistical Office of Slovak Republic [29].

There were no statistically significant differences in the development of the recovery of municipal waste produced by backward obtained organic matters, because *p* value reached a level of >0.1696 with a variability of regression model of about 16%. Figure 8 presents the results of the ANOVA analysis between selected regions in terms of backward obtained organic materials. The highest variability was recorded in the Bratislava region (BA) with the maximum rate of such recovery in 2018 (62,427.3 t) and lowest in 2012 (15,077.4 t) and with average annual recovery at the level 31,990.9 t·year^−1^, and conversely the lowest level in Prešov region with average annual recovery through backward obtained organic matters at 20,538.2 t·year^−1^, with the highest value of this kind of recovery in 2018 (39,877.3 t) and the lowest in 2013 (11,987.2 t).

## 4. Discussion

Results of this study have proven different states of the waste management in the regions of Slovakia. In view of this, the current “boom” in the European Union and in the world is now experiencing the concept of Circular Economy. It is an economic model, which in its title carries a concise policy of dealing with resources in circulation, constantly around, to the maximum possible use and closure of material flows. Its main concepts include the use of renewable energy, recycling, eco-innovation, eco-design, rental, sharing, and more [32].

In general, this means that even what had so far seemed to be a waste or could end up as waste, can be found, and currently is being actively sought for some other uses. If we speak in the context of sustainability, sustained economic growth, high competitiveness and new challenges, waste is a ready-made treasure—a new product [33,34].

Although the circular economy in the EU has caused a real buzz, the term itself is not entirely new. Especially when we say that, it goes hand in hand with issues like sustainable development, waste management, and the energy potential of waste.

**Figure 9:**
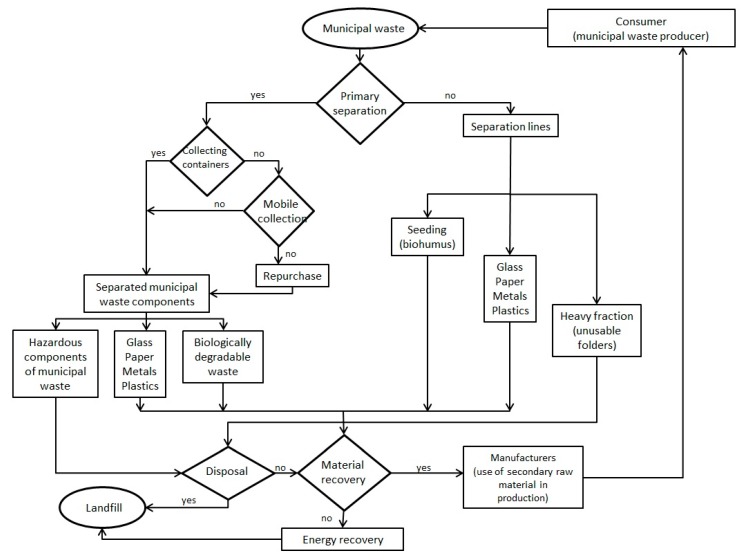
Proposal of implementation of Circular model into Municipal Waste Management. Source: Own processing.

In the area of resource efficiency, the European Commission took important initiatives during the years 2011–2015, with a first Circular Economy Package [11,18,35] further refined to become Circular Economy Action Plan [15]. Circular Economy (CE) has risen in popularity in recent years as a conceptual model to guide better use of natural resources and management of waste [36]. Results of this article have shown that policies must be implemented also on the regional level, because in Slovakia and probably in other countries, the differences between regions in waste treatment are significant. Waste management fees are set either by municipalities or by companies that further process waste, but these fees are different, with a very low separation rate in Slovakia—only around 16% and improving slowly [37]. Another problem in Slovakia is the low level of industry involvement in environmental activities, the positive news is the industry’s willingness to support eco-innovation [38]. The circular economy model is still not fully implemented in Slovakia, and it is playing its part in most western European countries [39] at even lower regional levels, a unifying model of circular economy would have to be designated. By synthesizing the results of the above-mentioned statistical analysis, we came to the conclusion that the implementation of the circular model in order to reduce interregional disparities in the management of produced municipal waste with an emphasis on waste recovery (both material and energy), which determines the integration of the principles of the circular economy should follow a clearly defined model of municipal waste recovery management, as shown in Figure 9 below.

We designed this model based on the results of the analysis but based on EU objectives and a thorough knowledge of waste management in the Slovak Republic. Slovakia is one of the weaker countries in terms of environmental efficiency, along with neighboring countries that are also EU Member States [16,40,41]. The implementation of such a proposed conceptual model is demanding in terms of personnel and financial resources. That is why in this paper, we have focused attention on the EU’s circular economy policy. Experts under the auspices of the EU can help with their knowledge to ensure a smooth process and possible elimination of several shortcomings. The successful implementation of this model could be the starting point for its implementation in other central European countries, in which it has still not been implemented to a significant extent. Challenges have support in modern strategies and tools. Internet of Things could bring more accurate policies and when implementing policies [42], it is necessary to build on new knowledge in industrial reverse logistics, such as synthesized in some works [43,44,45,46].

## 5. Conclusions

Waste recovering is today so genuine that it allows the generation of electricity and heat for households. Given the lack of natural resources, the original landfills will be a source of raw materials for the processing industry, because only 30% of the waste in landfills in several years will be lost to natural processes. All other waste has an industrial character and if we want it or not, plastics, papers, metals, or glass are the sources of raw materials. Innovative thinking might bring in future opportunities to explore and use raw materials from forgotten landfills. The innovative processes in waste economy for the last years started the revolution, which will continue. The development of innovation in waste economy will make waste as demand-oriented raw material which with each state has to think and handle as with raw material. It is the innovations that move the world and the market, and one of them is also circular economy.

The progress can be seen in the desired direction, but overall Slovakia is performing below-average on resource and waste management within the EU and is facing many challenges. However, as there is no uniform waste management in Slovakia, and there are different charges in each region, a different waste management policy is appropriate to introduce a uniform circular economy model and to create it in cooperation with both citizens and industry. Such a model could improve waste management, which is not as thorough in Slovakia as in other developed European countries. Legislative measures must also be ensured. Criteria for successful implementation should be established. It is Slovakia that could also use the structural funds to finance the implementation. The circular economy is not one of the government’s priorities and the related strategy documents do not clearly show the interconnection of the whole cycle. Another problem is the persistent double reporting of waste data, which, among other things, complicates the evaluation of the fulfillment of objectives. With regard to objectives, the challenge will be to achieve the required level of municipal waste recycling as well as to ensure the recycling of aluminum packaging if the target remains at the current relatively high level. The future research could focus on assessing the possibilities of implementations of such a model in central European countries, which in fact, have similar initial state as Slovakia. There can be models built which compare the waste production divided by industry sector and citizens. There are a lot of questions and problems which need to be addressed in the terms of circular economy.
